# Serum CYFRA 21-1 (cytokeratin-19 fragments) is a useful tumour marker for detecting disease relapse and assessing treatment efficacy in breast cancer

**DOI:** 10.1038/sj.bjc.6602074

**Published:** 2004-07-27

**Authors:** B Nakata, T Takashima, Y Ogawa, T Ishikawa, K Hirakawa

**Affiliations:** 1 Department of Surgical Oncology, Osaka City University Graduate School of Medicine, 1-4-3 Asahimachi, Abeno-ku, Osaka 545-8585, Japan

**Keywords:** CYFRA 21-1, cytokeratin-19 fragments, breast cancer, monitoring, carcinoembryonic antigen, carbohydrate antigen 15-3

## Abstract

The usefulness of serum CYFRA 21-1 (cytokeratin-19 fragments) in monitoring the recurrence of breast cancer and in evaluating therapeutic effects was studied retrospectively. The sera from 173 patients with primary breast cancer or recurrent disease were measured for CYFRA 21-1, carcinoembryonic antigen (CEA), and carbohydrate antigen 15-3 (CA 15-3) levels. The positive rates of serum CYFRA 21-1 for stage IV (*n*=12) or recurrent disease (*n*=26) were 83.3 and 84.6%, respectively, while those of serum CEA were 41.7 and 26.9%, and those of serum CA 15-3 were 83.3 and 34.6%. The elevated preoperative levels of serum CYFRA 21-1 decreased to normal levels after curative operation, whereas the levels remained abnormally high after noncurative operation. There was a significantly high frequency of recurrence in patients with elevated levels of serum CYFRA 21-1 preoperatively compared to those with normal levels of the marker preoperatively. The serum CYFRA 21-1 levels were well correlated with response to chemotherapy. The positive rate of serum CYFRA 21-1 alone was higher than that of an assay combining CEA with CA 15-3, in both primary and recurrent cases (28.8 *vs* 18.8 and 84.6 *vs* 46.2%, respectively). These observations suggest that serum CYFRA 21-1 may be a reliable marker of recurrence or therapeutic efficacy.

The clinical usefulness of serum tumour markers for breast cancer surveillance has not yet been established. Indeed, until sufficient data on this subject are obtained, the American Society of Clinical Oncology (ASCO) recommends against the routine use of even well-known antigens such as carcinoembryonic antigen (CEA) and carbohydrate antigen 15-3 (CA 15-3) after primary treatment or in monitoring responses to treatment (American Society of Clinical Oncology, 2001). For economic reasons, and to minimise radiation exposure, it is not possible to carry out the imaging studies, such as chest and abdominal computed tomography (CT) and bone scintigraphy, for follow-up and evaluation of therapeutic effect every 1–3 months. Therefore, it is clearly a worthwhile goal to find a reliable serum tumour marker with high sensitivity and high specificity for breast cancer. We previously reported that the positive rates of serum CYFRA 21-1, which reacts specifically with cytokeratin-19 fragments, in patients with stage IV or recurrent breast cancer were as high as those for CA 15-3 and superior to those for CEA. We also previously demonstrated that serum CYFRA 21-1 titre had prognostic predictive value in patients with breast cancer (Nakata *et al*, 2000). In this study, we measured serum CYFRA 21-1 titres prior to and after treatment in patients with breast cancer to observe the potential impact of the use of serum CYFRA 21-1 in monitoring for recurrence and in evaluating therapeutic efficacy.

## PATIENTS AND METHODS

### Patients

The Department of Surgical Oncology of Osaka City University Graduate School of Medicine first began to use a combination assay of serum CYFRA 21-1, CEA, and CA 15-3 for diagnosis and follow-up of patients with breast cancer in January 1999. Between January 1999 and May 2003, a total of 260 patients were treated for breast cancer at Osaka City University Hospital, but in 87 (33.5%) of these cases serum CYFRA 21-1 titres were not measured because the patients' doctors were not yet familiar with the combination assay system. These 87 cases were excluded from this analysis, leaving 173 patients in whom breast cancer was retrospectively investigated in the present study. Among these 173 patients, 119 were patients with primary tumours (untreated tumours) in whom sera were obtained from within 2 weeks prior to resection and at every 3–6 months after resection according to our follow-up system using the serum tumour markers CYFRA 21-1, CEA, and CA 15-3. These 119 patients also underwent routine imaging studies, including chest X-ray, abdominal ultrasonography/CT, and bone scintigraphy, every 6–12 months as part of our follow-up system. Among these 119 patients (median follow-up time after operation: 25.2 months; range: 3–54 months), 13 (10.9%) developed recurrent disease, and seven patients with high preoperative levels of serum CYFRA 21-1 happened to be measured in the early period of 7–14 days postoperation. In the remaining 54 patients (41 with primary breast cancer and, as in the fully assayed patients, 13 with recurrent cancer), sera were obtained only once due to the doctors' unfamiliarity with the combination assay system.

The Union Internationale Contre le Cancer (UICC) stages of the 160 patients with primary breast cancer were as follows: stage 0 (*n*=3), stage I (*n*=46), stage II (*n*=62), stage III (*n*=37), and stage IV (*n*=12) (Table 1

**Table 1:** Serum CYFRA 21-1, CEA, and CA 15-3 titres of 160 patients with primary breast cancer according to stage

**Variables**	**CYFRA 21-1 (ng ml^−1^)**	**CEA (ng ml^−1^)**	**CA 15-3 (U ml^−1^)**
Cutoff value	2.0	6.5	30
			
*UICC staging*	Median (range)	Median (range)	Median (range)
0 (*n*=3)	0.8 (0.2–1.8)	1.4 (1.0–3.6)	11.3 (6.8–12.3)
I (*n*=46)	1.3 (0.3–3.0)	2.8 (0.8–9.2)	12.6 (6.5–30.8)
II (*n*=62)	1.4 (0.5–6.2)	3.0 (0.9–29.3)	16.2 (5.7–141)
III (*n*=37)	1.3 (0.5–23)	2.4 (0.6–48.3)	15.9 (5.4–165)
IV (*n*=12)	4.4 (1.2–122)	4.7 (1.1–24.7)	76.3 (14.2–4470)
			
Kruskal–Wallis test	*P*<0.0001	*P*=0.1285	*P*<0.0001
			
Mann–Whitney *U*-test	*P*-value	*P*-value	*P*-value
Stage 0 *vs* I–IV	0.2568	0.1550	0.0691
Stage 0–I *vs* II–IV	0.0046	0.0808	0.0001
Stage 0–II *vs* III–IV	0.0003	0.5038	0.0001
Stage 0–III *vs* IV	<0.0001	0.0676	<0.0001

CEA=carcinoembryonic antigen; CA=carbohydrate antigen.

UICC=Union Internationale Contre le Cancer.

). The staging was based on the complete pathological findings and imaging studies for each patient. As described above, the total number of recurrent cases was 26, among which 13 patients were measured for serum CYFRA 21-1 serially and 13 patients were assessed only once. In 14 patients, a change in serum CYFRA 21-1 levels was observed during chemotherapy. Among them, four patients were treated neoadjuvantly for primary tumours and distant metastases (lung (*n*=2), liver (*n*=1), and bone (*n*=1)). The other 10 patients were treated for recurrent metastases (bone (*n*=3), bone+liver (*n*=3), lung (*n*=2), lung+bone (*n*=1), and lymph node (*n*=1)). All lesions except bone metastases were measurable by CT or ultrasonography, and bony lesions were evaluable by bone scintigraphy. Objective responses were classified according to World Health Organisation criteria (World Health Organization, 1979).

### Measurement of tumour markers

Serum CYFRA 21-1 was measured by a solid-phase immunoradiometric assay based on the two-site sandwich method using a CYFRA 21-1 kit (CIS Biointernational, Gif Yvette, France) (Sarwar *et al*, 1994). The measurement of serum CEA was completed in a counting immunoassay. Serum CA 15-3 was measured by a two-step sandwich immunoradiometric assay. The cutoff values of CYFRA 21-1, CEA, and CA 15-3 recommended by the manufacturers were 2.0 ng ml^−1^, 6.5 ng ml^−1^, and 30 U ml^−1^, respectively.

### Measurement of estrogen receptor (ER) and progesterone receptor (PgR)

ER status and PgR status were determined by an enzyme-linked immunoassay, and tumours with more than 5 fmol mg^−1^ protein were considered to be positive.

### Statistical analysis

Statistical analysis was performed using nonparametric methods. The Kruskal–Wallis one-way analysis was used for multiple comparison of the UICC staging groups 0–IV. The Mann–Whitney *U*-test was used for comparisons between pairs of independent groups divided according to UICC staging, ER status, and PgR status. The *χ*^2^ test (Fisher's exact method) was used to compare between variables. *P*-values <0.05 were considered to be statistically significant.

## RESULTS

### Serum CYFRA 21-1 titre according to ER and PgR status

The serum CYFRA 21-1 levels of the 87 ER-positive tumours were not different from those of the 68 ER-negative tumours (median values: 1.3 *vs* 1.45 ng ml^−1^, respectively; *P*=0.0679). The serum CYFRA 21-1 levels of the 77 PgR-positive tumours were not different from those of the 78 PgR-negative tumours (median values: 1.4 *vs* 1.4 ng ml^−1^, respectively; *P*=0.6762).

### Serum tumour marker level in each stage for primary breast cancer

The serum CYFRA 21-1, CEA, and CA 15-3 titres of all 160 primary cases are shown in Table 1. The serum CYFRA 21-1 and CA 15-3 levels were significantly different among stages 0–IV (the Kruskal–Wallis one-way analysis; both, *P*<0.0001), although the serum CEA levels were not. Table 1 demonstrates the difference between the serum levels of the two groups according to UICC staging in each tumour marker examined based on the results of the Mann–Whitney *U*-test. The serum CEA levels were not significantly different between any two groups. The serum CYFRA 21-1 and CA 15-3 levels were significantly different between all pairs of groups except 0 *vs* I–IV, although the *P*-values for CA 15-3 were a little smaller than those for CYFRA 21-1.

Figure 1

**Figure 1:**
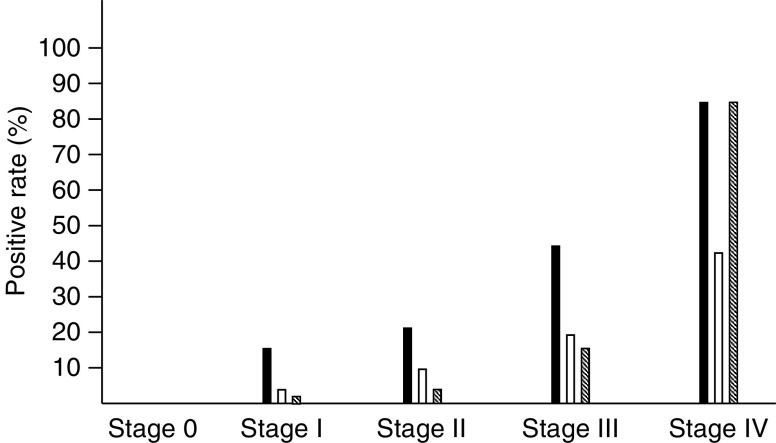
Comparison of the positive rates of serum CYFRA 21-1 (solid bar), CEA (open bar), and CA 15-3 (striped bar) at each stage.

compares the positive rates of serum CYFRA 21-1, CEA, and CA 15-3 for each stage grouping. No tumour marker was elevated for stage 0. Among these three markers, CYFRA 21-1 had the highest rate of positivity in stages I–IV. At stage IV, 10 of 12 patients (83.3%) showed elevated levels of both serum CYFRA 21-1 and CA 15-3.

### Serum tumour marker level for recurrent breast cancer

**Figure 2:**
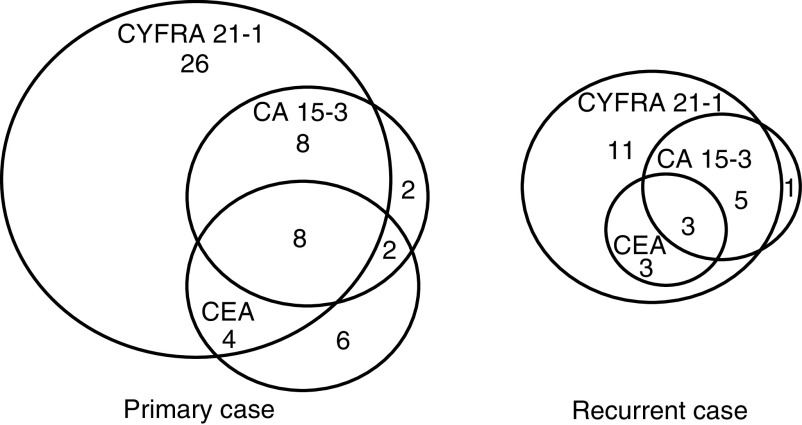
Distribution of patients with elevated levels of serum CYFRA 21-1, CEA, and CA 15-3 in primary and recurrent breast cancer. The number in each area is the number of patients with elevated levels of tumour markers.

Among the 26 recurrent cases, 84.6% (22 of 26) were positive for serum CYFRA 21-1, whereas 26.9% (six of 26) and 34.6% (nine of 26) were positive for serum CEA and CA 15-3, respectively (Figure 2

). When the *χ*^2^ test was used to examine the rate of positivity for each of the three tumour markers in the sera of patients with recurrence, significant differences between CYFRA 21-1 and CEA (*P*<0.0001) and between CYFRA 21-1 and CA 15-3 (*P*<0.0003) were observed. However, there was no difference between CEA and CA 15-3 (*P*=0.3803).

### Changes in serum CYFRA 21-1 levels by resection

**Figure 3:**
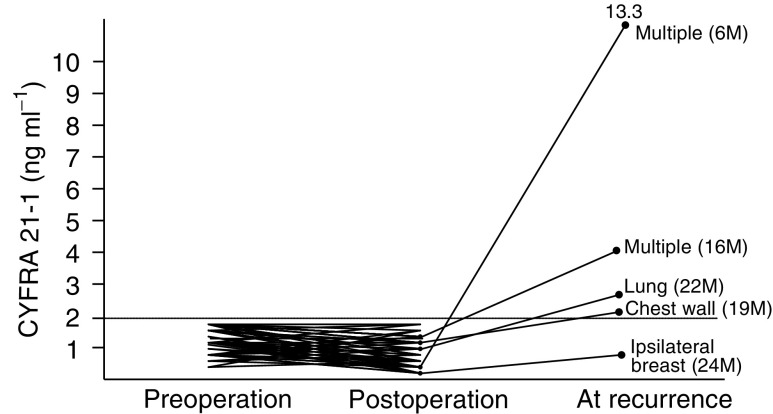
Change of serum CYFRA 21-1 titres of patients within normal levels at preoperation. The recurrent site is demonstrated for each case, and the recurrent time after operation is shown within parentheses.

**Figure 4:**
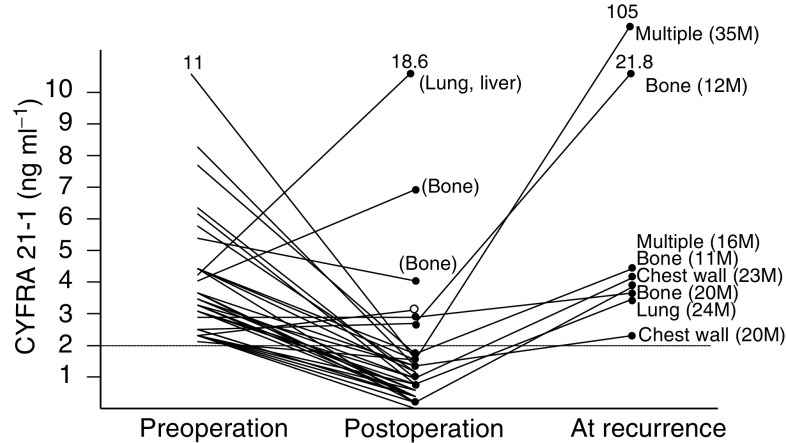
Changes in serum CYFRA 21-1 titres of patients with elevated levels at preoperation. Residual sites are within parentheses. A recurrent site is demonstrated for each case, and the postoperation time until recurrence is shown within parentheses. The open circle indicates a patient with an elevated level of serum CYFRA 21-1 at postoperation, who nevertheless has shown no evidence of relapse.

The sera from 119 patients with primary tumours were measured pre- and postoperatively. All 84 patients with negative preoperative serum CYFRA 21-1 titres had normal levels postoperatively. However, among the five such patients who developed a later recurrence, four showed elevated levels of serum CYFRA 21-1. One of these patients, who had an ipsilateral recurrence after breast conservation therapy, had a normal serum CYFRA 21-1 titre (Figure 3

). Among the 35 patients with elevated levels of serum CYFRA 21-1 preoperatively, 29 (82.9%) showed a decrement to within the normal range postoperatively. Among these 29 patients, six (20.7%) developed recurrent tumours by 11–35 months postoperatively and all showed elevated levels of serum CYFRA 21-1. Of the six patients in whom the serum CYFRA 21-1 levels continued to be elevated postoperatively, three underwent noncurative operation for lung, liver, or bone metastases; two developed bony recurrences at 12 and 20 months after operation; and the sixth patient has so far shown no evidence of recurrence after curative operation (Figure 4

).

Among the 13 recurrent patients whose serum CYFRA 21-1 was monitored serially, two patients showed elevated serum CYFRA 21-1 titres before the detection of metastatic lesions by imaging studies. Both of these patients developed liver metastases and showed elevated levels of CYFRA 21-1 at 3 or 4 months, respectively, before the detection of tumours by CT scan. In the other 11 patients, the dates of tumour detection by imaging studies were almost identical to the dates of detection by elevation of serum CYFRA 21-1. The reason for this may have been related to our follow-up system, in which the measurement of tumour markers and imaging studies were simultaneously ordered. Another possible reason may have been that, in all patients, imaging studies were immediately performed whenever the serum CYFRA 21-1 was found to be elevated.

**Figure 5:**
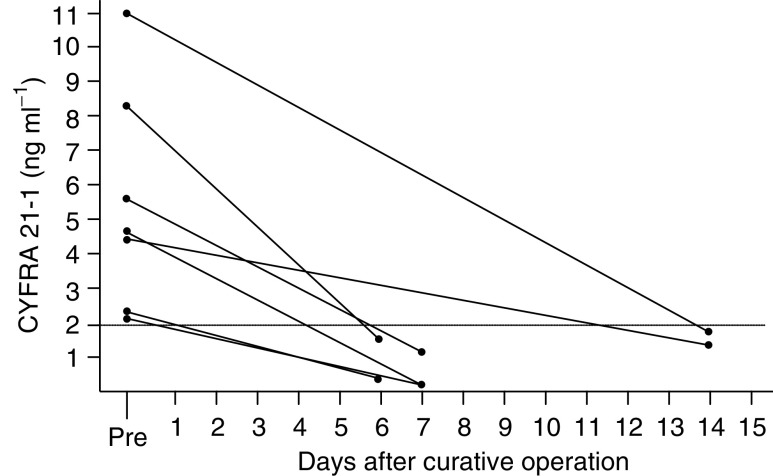
The decrement of serum CYFRA 21-1 titre after curative operation.

The sera from seven patients with elevated serum CYFRA 21-1 levels (median: 4.5 ng ml^−1^; range: 2.3–11 ng ml^−1^) preoperatively were measured within 2 weeks after operation. All the serum CYFRA 21-1 titres decreased to within the normal range during this period (Figure 5

). These data demonstrate how quickly the marker decreased in the sera of these patients.

### Relation between serum CYFRA 21-1 levels and recurrence

Among the 119 primary breast cancer patients with pre- and postoperative serum CYFRA 21-1 data, 116 patients underwent potentially curative operation. These data were analysed to examine the association between serum CYFRA 21-1 level and disease recurrence. As shown in Table 2

**Table 2:** Comparison of frequency of disease recurrence after potentially curative operation according to pre- and postoperative levels of serum CYFRA 21-1

	**Serum CYFRA 21-1 (ng ml^−1^)**		**Number of patients**		
**Group**	**Preoperation**	**Postoperation**	**Recurrence**	**No recurrence**	**Total**
A	<2	<2	5 (6.0%)	79	84
B	>2.1	<2	6 (20.7%)	23	29
C	>2.1	>2.1	2 (66.6%)	1	3

According to serum CYFRA 21-1 levels at pre- and postoperation, the patients were divided into groups A, B, and C.

Frequencies of recurrences were different between A and B (*P*=0.0312), A and C (*P*=0.0162), and A and B+C (*P*=0.007), but not B and C (*P*=0.1468), examined by *χ*^2^ test (Fisher's exact method).

, the patients in whom serum CYFRA 21-1 levels were elevated both pre- and postoperatively had the greatest frequency of recurrence. In patients in whom the serum CYFRA 21-1 levels were elevated preoperatively and normal postoperatively, the frequency of recurrence was 20.7%. Only 6% of the patients with normal levels of serum CYFRA 21-1 preoperatively developed recurrences. The frequency of recurrence in patients with elevated levels of serum CYFRA 21-1 preoperatively was significantly higher than that in patients with preoperatively normal levels of the marker (*P*=0.007).

### Changes in serum CYFRA 21-1 levels after chemotherapy

Figure 6

**Figure 6:**
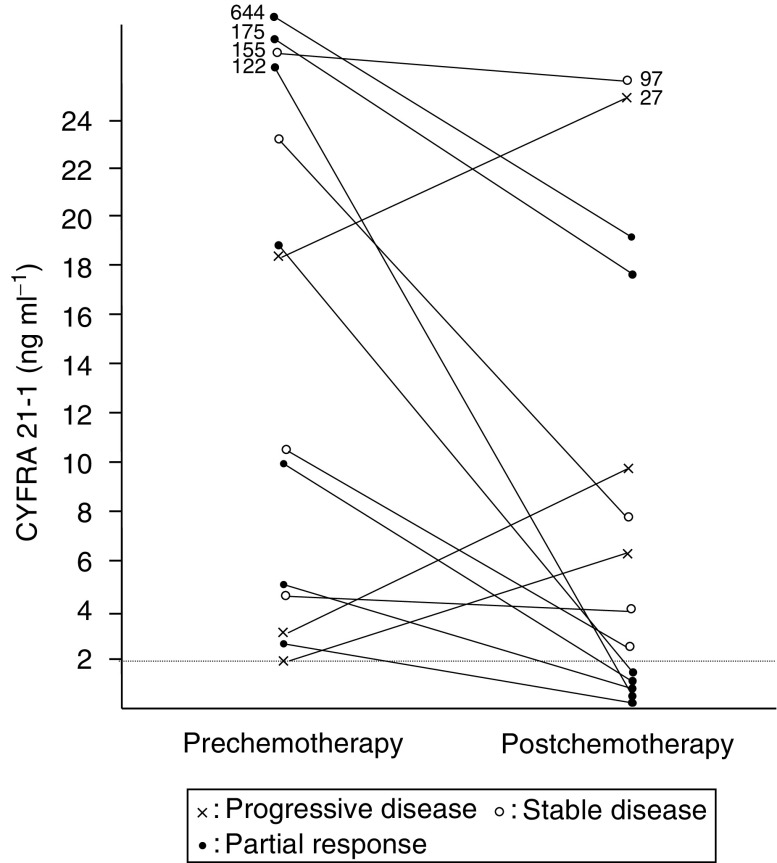
Change in serum CYFRA 21-1 titres after chemotherapy.

demonstrates the changes in serum CYFRA 21-1 levels before and after chemotherapy. These 14 patients were administered intensive chemotherapeutic regimens, such as CAF (cyclophosphamide+adriamycin+5-fluorouracil), FEC (5-fluorouracil+epirubicin+cyclophosphamide), or taxane with or without herceptin. The serum CYFRA 21-1 levels in five of seven patients with postoperative recurrence decreased to within the normal range after chemotherapy, and those of the other two patients with extremely high levels (175 and 644 ng ml^−1^) prior to chemotherapy decreased to approximately 3 and 10% of the prechemotherapy levels after chemotherapy, respectively. In the four patients with stable disease, chemotherapy reduced the serum CYFRA 21-1 levels to approximately 20–90% of the prechemotherapy levels. In the three patients with progressive disease, the serum CYFRA 21-1 levels were increased by 150–400% following chemotherapy.

### Combination assay of tumour markers for breast cancer

Figure 2 demonstrates the positive case number for each tumour marker. Among the 160 patients with primary tumours, 104 (65%) showed normal serum levels of all tumour markers and 46 (28.8%) had elevated CYFRA 21-1 levels. Also among the primary cases, 26, six, and two patients showed elevated levels of serum CYFRA alone, CEA alone, and CA 15-3 alone, respectively. The rates of positivity for CYFRA 21-1, CEA, and CA 15-3 among the 160 primary tumour cases were 28.8, 12.5, and 12.5%, respectively. The rate of positivity for all three markers in the combined assay was 35%; CYFRA 21-1 combined with CEA was 33.8%; CYFRA 21-1 with CA 15-3 was 31.3%; and CEA with CA 15-3 was 18.8% among these 160 patients. Among the 26 patients with recurrent tumours, 22 (84.6%) had elevated CYFRA 21-1 levels. Three of the 26 recurrent patients (11.5%) showed normal serum levels of all tumour markers. These marker-negative tumours occurred in the lung, the axillary lymph node, or the ipsilateral breast, respectively. Among recurrent cases, 11, 0, and one patients showed elevated levels of serum CYFRA alone, CEA alone, and CA 15-3 alone, respectively. The positive rates of CYFRA 21-1, CEA, and CA 15-3 for recurrent tumours were 84.6, 23.1, and 34.6%, respectively. In recurrent cases, the positive rate of the combined assay using all three markers was 88.5%; CYFRA 21-1 combined with CEA was 84.6%; CYFRA 21-1 with CA 15-3 was 88.5%; and CEA with CA 15-3 was 46%.

## DISCUSSION

It has been well demonstrated that breast cancer cells express fragments of cytokeratin-19 (Moll *et al*, 1982; Brotheric *et al*, 1998; Sheard *et al*, 2002), which is one of the various kinds of cytokeratins comprising the intermediate filaments of the cytoskeleton (Moll *et al*, 1982). Serum fragments of cytokeratin-19 can be detected using anti-CYFRA 21-1 antibody (Pujol *et al*, 1993). Most of the epitopes that are detectable by clinically useful tumour markers such as CEA, CA 15-3, CA 19-9, and alpha-fetoprotein are glycoproteins shed from the cell surface. CYFRA 21-1 is unique in that its epitope is a polypeptide, which is most likely released following cell death (Stieber *et al*, 1993; Sheard *et al*, 2002). Elevated levels of serum CYRFA 21-1 titres have been observed in various malignancies, especially in lung cancer (Molina *et al*, 1994). Healthy individuals with an abnormal level of serum CYFRA 21-1 are quite rare (Molina *et al*, 1994; Nakata *et al*, 1996; Giovanella *et al*, 2002). Patients with nonmalignant disease also showed almost no elevation of serum CYFRA 21-1, except in cases of cirrhosis, renal failure, or infectious lung disease. In previous studies, 20–30% of patients with one of these three benign diseases showed elevated levels of serum CYRFA 21-1 (Molina *et al*, 1994; Nakata *et al*, 1996). However, little is known about this tumour marker for breast cancer (Molina *et al*, 1994; Nakata *et al*, 2000; Giovanella *et al*, 2002; Rodriguez *et al*, 2002).

Our previous report demonstrated that, in patients with stage IV or recurrent disease, the positive rate of serum CYFRA 21-1 was as high as that of CA 15-3 and superior to that of CEA (Nakata *et al*, 2000). In this study, we measured serum CYFRA 21-1 in a new series of patients, and the results for primary breast cancer were the same, except that the positive rate of serum CYFRA 21-1 for recurrent disease was higher than that of serum CA 15-3 (Figures 1 and 2). Rodriguez *et al* (2002) examined the serum CYFRA 21-1, CEA, and CA 15-3 titres in their 40 patients with metastatic breast cancers and found that CYFRA 21-1 was a sensitive tumour marker for breast cancer when compared with CEA or CA 15-3, supporting our results. However, contrary to our results, Giovanella *et al* (2002) reported that serum CYFRA 21-1 was less accurate for the evaluation of primary and recurrent breast cancer than serum CA 15-3. The low diagnostic value of CYFRA 21-1 in breast cancer reported by Giovanella *et al* should be due to their higher serum CYFRA 21-1 cutoff value (3.3 ng ml^−1^) compared to our cutoff value (2.0 ng ml^−1^), although both studies used the same CYFRA 21-1 kit (CIS Biointernational). Our cutoff value was decided by the manufacturer's recommendation, while Giovanella *et al* used the cutoff value originally selected for lung cancer and benign lung diseases. Giovanella *et al* described that they could not employ a lower cutoff in a breast disease setting, because 5% of the healthy individuals in their series showed a high level above 3.3 ng ml^−1^.

In our study, the serum marker levels among each stage were examined by nonparametric analyses, and CYFRA 21-1 and CA 15-3 were correlated significantly with stage, although CEA was not (Table 1). However, as described in our previous report (Nakata *et al*, 2000) and as shown in this report, the measurement of serum CYFRA 21-1 cannot be a potential screening test due to the low positive rate for early breast cancer. We also demonstrated in a previous report (Nakata *et al*, 2000) that serum CYFRA 21-1 has not only a high positive rate for breast cancer but also high specificity for breast cancer, that is, 22 patients with benign mammary disease did not show elevated levels of serum CYFRA 21-1. Molina *et al* (1994) also reported that only 4% (one of 25) of patients with benign mammary disease had elevated levels of serum CYFRA 21-1.

The time course of serum CYFRA 21-1 titre during treatment has not been investigated previously. We observed 119 patients with primary tumour at pre- and postoperation. We surveyed these patients and found that 13 of them had had a recurrent tumour. Among the 35 patients with elevated levels of serum CYFRA 21-1 at preoperation, only six patients had abnormal levels of this tumour marker at postoperation. Among the six cases, the high levels in three cases were attributable to residual tumours, while the high levels in two cases were probably due to micrometastasis (Figure 4). These results suggest that serum CYFRA 21-1 may be a good marker for surgical curability. Moreover, we found that the high serum titre of CYFRA 21-1 at preoperation decreased to a normal level within 2 weeks after curative operation (Figure 5). All but one of the patients who developed recurrent disease showed elevated levels of CYFRA 21-1, and the one exception had an ipsilateral recurrence after breast conservation therapy (Figures 3 and 4). These results suggest that serum CYFRA 21-1 might be a reliable marker of recurrent disease. Among the 84 patients with normal levels of serum CYFRA 21-1 preoperatively, five developed recurrences and four of these demonstrated elevated levels of this tumour marker. This indicates the potential usefulness of the marker for monitoring disease relapse, even in cases with normal levels of serum CYFRA 21-1 at preoperation. Moreover, the preoperative abnormal levels of CYFRA 21-1 were significantly related to higher incidence of the disease relapse (Table 2).

The leading time of a tumour marker elevation before manifestation of relapse by imaging studies is an important subject. However, our retrospective study demonstrated only two anecdotal cases in which the leading times of serum CYFRA 21-1 were 3–4 months. To investigate the leading time, a prospective study may be required, in that serum CYFRA 21-1 should be measured monthly.

The chemotherapeutic effects of CYFRA 21-1 correlated well with the changes in serum levels of CYFRA 21-1 between pre- and postchemotherapy (Figure 6). Partial response efficacy may be assessed by changes in serum CYFRA 21-1 when the elevated titre decreases to 10% or less of the preoperative value, or when the normal range is reached at postchemotherapy.

We observed that the positive rates of serum CYFRA 21-1 alone in primary and recurrent cases were superior to those of the assay combining CEA with CA 15-3. The assays combining CYFRA 21-1 with CEA or CA 15-3 increased the positive rate modestly, increased the positive rate by 5 or 2.5% in primary cases, and 0 or 3.9% in recurrent cases, respectively (Figure 2). These findings indicated that serum CYFRA 21-1 may be useful even in a single assay, when a combination assay using two or more tumour markers is not feasible economically.

In conclusion, the measurement of serum CYFRA 21-1 may be useful for detecting disease relapse and for assessing surgical and chemotherapeutic efficacy. However, the patient number in this study was too small to draw any definitive conclusions. Further prospective studies using greater numbers of patients are required before serum CYFRA 21-1 can be recommended for routine clinical use.
